# Will the next pandemic be caused by coronavirus again? An interview with George Fu Gao

**DOI:** 10.1093/nsr/nwae477

**Published:** 2024-12-27

**Authors:** Weijie Zhao

## Abstract

*For Chinese people, 2024 has been a good year. The shadow of the COVID-19 pandemic has dissipated; social activities and domestic travels have largely returned to normal; and they enjoyed the unity and enthusiasm of the Paris Olympic Games together with the whole world. However, there is a lingering thought in everyone’s mind that there may be ‘a next pandemic’ coming sometime in the future. ‘Be prepared for danger in times of peace’ is a wisdom that never goes out of style. National Science Review (NSR) conducted an interview in September 2024 with Prof. George Fu Gao, the former Director General of the Chinese Center for Disease Control and Prevention, an Academician of the Chinese Academy of Sciences, and a top scientist in pathogenic microbiology and immunology. In this interview, Gao talked about the possibilities of ‘the next pandemic’ from a scientific perspective, and calls on the world to maintain openness, sharing and collaboration in research and action related to global health*.

## Teaser

Former Director General of China CDC George Fu Gao talked about the 'the next pandemic' from a scientific perspective, and calls on the world to collaborate for global health.

## THE NEXT PANDEMIC

***NSR:*** Now there is a general belief that there will be a ‘next pandemic’. When might it come?

***Gao:*** The next pandemic will definitely come, but it is hard to predict when. It may come tomorrow, and it may come decades later that I will not see it in my lifetime.

The Earth was born 4.6 billion years ago; the microorganisms such as viruses and bacteria, including pathogens, were born 3.3–2.6 billion years ago; while *Homo sapiens* was born only about 200–40 thousand years ago. Therefore, it is appropriate to say that viruses and bacteria are the original inhabitants of the Earth, and human beings have violated their living space as latecomers. The original inhabitants may become ‘angry’ with us and ‘beat us up’ at any time, bringing an epidemic, or even a pandemic.

***NSR:*** Which virus may cause the next pandemic?

***Gao:*** Any virus is possible, but the most likely ones are definitely respiratory viruses, because compared with fecal-oral or direct contact transmission, respiratory transmission is the most difficult to prevent. Everyone has to breathe, and we cannot control the quality of the air you breathe at anytime and anywhere. For fecal-oral transmission, we can thoroughly disinfect the food and water, but we are not able to do that for air. By the way, the greatest achievement of human's public health construction is that we have solved the problem of water source cleanliness, and thus controlled cholera and many other fecal-oral transmitted diseases in many countries and areas, including China. But we have to see that the water source problem has not been solved in Africa and some other regions, and such diseases are still transmitting in these areas.

Back to the question, the next pandemic is most likely to be caused by a respiratory virus, but which virus will it be? Scientists’ answer is that the most likely viruses are still influenza viruses or coronaviruses. No other virus has shown a greater potential than these two categories.

**Figure 1:**
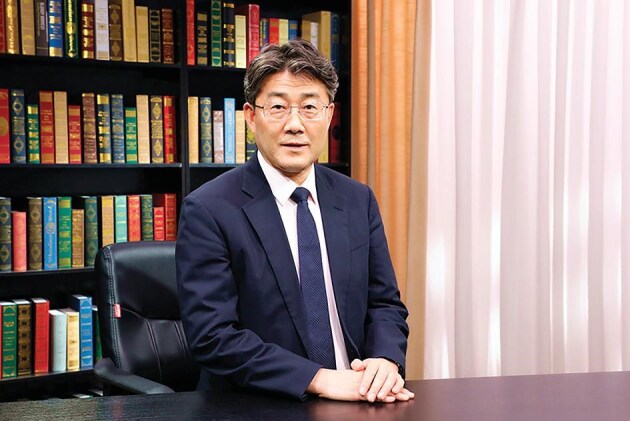
Prof. George Fu Gao is the former Director General of the Chinese Center for Disease Control and Prevention, and a top scientist in pathogenic microbiology and immunology. *(Courtesy of Prof. Gao)*

On 18 October 2019, I participated in the Event 201 in the USA, which was an international exercise for the outbreak of a pandemic, organized by Johns Hopkins University together with Bill & Melinda Gates Foundation and World Economic Forum. The imaginary pandemic in this exercise was called CAPS, coronavirus associated pulmonary syndrome: almost COVID-19. Some people questioned: ‘Why you had this exercise just before the outbreak of COVID-19? Isn't it too coincidental?’ There is no coincidence. We scientists have been predicting for a long time that the next pandemic will be influenza or coronavirus. The Global Preparedness Monitoring Board (GPMB), for which I was a member, predicted in its annual report released at the end of 2019, which was drafted before the emergence of COVID-19, that the pathogen that may cause a pandemic in the future

The most likely viruses are still influenza viruses or coronaviruses.

—George Fu Gao

is either influenza or coronavirus. This judgment has long been the consensus of the community.

Why did we think that coronavirus could bring a global pandemic? First, the coronaviruses that infect people mix with the influenza viruses every year to bring seasonal cold. These two kinds of respiratory virus can easily spread and are able to cause large-scale outbreaks. Second, there has been a SARS (severe acute respiratory syndrome) epidemic caused by coronavirus in 2003. The SARS epidemic was very dangerous, but fortunately the virus did not adapt well to humans and did not become a big world pandemic. After killing about 800 people worldwide, it gradually subsided. At the beginning of the COVID-19 pandemic, many people thought that COVID-19 would end as quickly as SARS and would not become a serious global problem, which turned out to be a misjudgment.

For the abovementioned reasons, we have long known that influenza viruses and coronaviruses are the pathogens most likely to cause global pandemics. It was the answer before COVID-19, and is still the answer now.

***NSR:*** What will be the severity of the next outbreak?

***Gao:*** This is not easy to predict. Even at the beginning of the COVID-19 pandemic, few people could predict that it would develop into such a huge problem.

We cannot exclude the possibility of another serious global pandemic like COVID-19, because such things have happened repeatedly in history. Before COVID-19, both plague and influenza had seriously outbroken and endangered human society.

But from a professional point of view, as people have been widely exposed to coronavirus in this pandemic and have gained some basic immunization, it is unlikely that such a large-scale coronavirus pandemic will occur again in the future. One indirect evidence is that the 2009 influenza epidemic was not very serious—an important reason for that is people had been exposed to influenza viruses every year and already had basic immunization. This is why we keep encouraging people, especially those of elderly and immune compromised, to be vaccinated with flu vaccine every year to ‘expose’ to the ‘dead’ virus to gain certain immunity.

Of course, humans can never predict the evolution of viruses, so we cannot rule out the possibility that new viruses will emerge and bring large-scale pandemics again. These two viruses have their own characteristics, as influenza virus can reassort with different gene segments and coronavirus can recombine with gene pieces from different coronavirus strains.

***NSR:*** On 14 August 2024, the World Health Organization (WHO) declared the mpox (previously known as monkeypox) outbreak as a Public Health Emergency of International Concern (PHEIC) for a second time. How will the mpox epidemic develop?

***Gao:*** On 23 July 2022, WHO declared the mpox outbreak as a PHEIC for the first time. On 11 May 2023, it was announced no longer a PHEIC. And on 14 August 2024, it was the second time that the mpox was declared a PHEIC.

The main reason for this second announcement is that in the Democratic Republic of Congo and some other African regions, mpox has recently spread fast and caused many cases, and more importantly, some scientists suspect that the latest mpox virus strain has become able to be transmitted through the air. This remains a suspicion to be confirmed.

In China, since the first mpox case imported from Germany reported in September 2022, mpox cases keep appear intermittently until now. The main virus strain spread in China belongs to Clade II, while the strain spread in Africa and suspected to be respiratory transmitted is Clade Ib.

At the beginning, mpox was mainly transmitted among men who have sex with men (MSM). But now it has spread to the general population through sexual transmission. In the future, if it further adapts to the population, or respiratory transmission does occur, it may indeed become a larger outbreak.

***NSR:*** WHO recently updated the list of pathogens that may trigger the next epidemic or pandemic. What is the significance of this list?

***Gao:*** This list is instructive, not for us scientists in the field of infectious diseases—we know which pathogens are dangerous no matter there is a list or not, but for the public and the policymakers.

For the public, the list can help them distinguish between true and false information when the outbreak comes, and prevent them to be trapped by the ‘information pandemic’, i.e. infodemic. For policymakers, the list can instruct them to prepare for the next outbreak. It tells them the funding should go to the basic research of the viruses on the list, including coronavirus, influenza virus, mpox virus, Nipah virus, Hendra virus and so on.

***NSR:*** In the next pandemic, will we perform better than we did during the COVID-19? In other words, what legacy has COVID-19 left us?

***Gao:*** We have made no major mistakes in the overall response to COVID-19. We worked as fast and as hard as we can to simultaneously perform non-pharmacological interventions

What we have not done well enough in the COVID-19 pandemic—I mean data collection and data sharing—I don't think we will do any better in the next pandemic.

—George Fu Gao

(NPI), patient care, and drug and vaccine development. A number of measures have been taken to fight the virus in an all-round way.

What we have not done well enough in the COVID-19 pandemic—I mean data collection and data sharing—I don't think we will do any better in the next pandemic. Just as philosopher Hegel said: ‘The lesson we learnt from history is that we do not learn from history.’ The pandemic has not made the world more united, and the disease control systems in many countries have not been better organized or become more efficient. The ‘legacy’ of COVID-19 may not be passed on.

But as scientists, we still have to call for data openness and data sharing. Infectious diseases are not a problem of any single country, but a challenge of the whole world. With one airplane taking off and landing on the other side of the planet, the virus can cross mountains and seas and spread all over the world. No matter how the international relations change, human beings have to keep openness, sharing and collaboration in at least two areas: human health and climate change. These are the challenges related to the survival of mankind and we must unite to face together.

***NSR:*** What should scientists and government agencies do to prepare for the next pandemic?

***Gao:*** In 2018, I published a commentary in *Cell* [1], in which I wrote that facing emerging infectious diseases, there are only two things that humans can do. The first is to invest in basic research on the infectious diseases, so that we will be ready to develop drugs and vaccines as soon as an epidemic comes. The second is to keep watching on the potential pathogens. We need to build a better monitoring system so that we can acknowledge the outbreak and the dynamics of the viruses with no time lost. In addition, we will continue to call for data sharing and try to build a platform for global health data sharing.

## OTHER ISSUES RELATED TO INFECTIOUS DISEASES

***NSR:*** Is it a natural law that viruses become more transmissible and less pathogenic during their continuous mutation? What does this mean for human response to the epidemics?

***Gao:*** Yes, this is a natural phenomenon. For humans, it means that, as WHO called on at the beginning of the COVID-19 outbreak, we should possess tolerance and resilience. As I

said at the beginning of this interview, viruses are the original inhabitants of the Earth, so the war between humans and viruses is definitely to be long-lasting. In history, humans have not been able to eliminate any virus except smallpox and rinderpest. Facing the viruses, we need tenacious resilience to pass through the long-lasting war, and learn to live together with the viruses with great tolerance. In fact, many countries have shown such resilience during the COVID-19 pandemic.

Scientists have the responsibility to speak out during the outbreaks, and the government should support and encourage them.

—George Fu Gao

***NSR:*** Scientists have not found the definite source of SARS-CoV-2. Will we find the answer?

***Gao:*** It is very likely that there will not be a definite conclusion for the source of SARS-CoV-2. In collaboration with Jun Liu (Chinese Center for Disease Control and Prevention) and others, we published an article in *Nature* [2], which scientifically confirmed that the Huanan Seafood Market in Wuhan might not be the initial source of the pandemic, and the raccoon dogs sold in this market are not the animal hosts that directly transmitted the virus from animals to humans. The data relevant to this study is open accessed. This work overturned the suspicion of many scientists. But this is science, and we have to search for answers by science.

If the Huanan Seafood Market and the raccoon dogs are not the source, where does the virus come from? We may never have a definite answer for this question. Actually, we do not really know where the human immunodeficiency virus (HIV) comes from even today. The HIV is said to originate in wild primates in central Africa, but actually, the original virus in those monkeys is very different from the HIV virus first discovered in the USA. It will take at least decades for the virus to evolve from the former to the latter. What happened during these intervening decades? Or is HIV really derived from this wild virus or not? Are there other possibilities? We still have no answer.

For a specific virus, there may be no definite conclusion about its source, but I still believe that scientists’ work to trace them is valuable, because that is the only possible way to find the truth.

***NSR:*** Is traditional Chinese medicine (TCM) and other traditional medical approaches effective for treating COVID-19? Do we know how to study and develop TCM using modern scientific methods?

***Gao:*** I have always believed that there is no ‘traditional Chinese medical science’ or ‘Western medical science’. There is only one discipline, medical science, which is a highly applied science dedicated to curing diseases and saving lives.

People often talk about the modernization of TCM. But I think that the ‘scientification of TCM’ may be a more appropriate term. We need to apply various modern sciences to the research and development of TCM.

Many Chinese scientists are already trying to do this. My group has also published several articles in this field [3,4]. We proved that the TCM decoction does have antiviral effects, and successfully extracted from the decoction active ingredients that have antiviral and anti-inflammatory functions. Such research works are beneficial to the scientification of TCM, and the development of medical science.

***NSR:*** How should scientists communicate with the public during the outbreaks?

***Gao:*** Scientists have the responsibility to speak out during the outbreaks, and the government should support and encourage them. There are two ways for scientists to share their findings and opinions: the first is to directly communicate with the public, and the second is to publish academic articles in the scientific journals to share their research data with the world. Both approaches need to be protected and encouraged.

***NSR:*** Will we defeat HIV, and how?

***Gao:*** Now it seems that there is little hope of developing an effective HIV vaccine. We need breakthroughs in immunology to create new opportunities.

## Notes

Weijie Zhao is an NSR news and science editor based in Beijing.
